# Case of Hemolysis, Elevated Liver Enzymes, and Low Platelet Count (HELLP) Syndrome Complicated by Sepsis: Challenges in Diagnosis and Management

**DOI:** 10.7759/cureus.104237

**Published:** 2026-02-25

**Authors:** Hui Li, Meirong Du, Xia Li, Xiaolan Liu

**Affiliations:** 1 Department of Obstetrics and Gynecology, Ordos Central Hospital, Inner Mongolia Autonomous Region, Ordos, CHN; 2 Department of Obstetrics and Gynecology, Obstetrics and Gynecology Hospital, Tongji University, Shanghai, CHN; 3 Department of Hematology, Ordos Central Hospital, Inner Mongolia Autonomous Region, Ordos, CHN

**Keywords:** coagulopathy, endothelial dysfunction, hellp syndrome, multidisciplinary management, obstetric emergency, sepsis

## Abstract

Hemolysis, Elevated Liver Enzymes, and Low Platelet Count (HELLP) syndrome, complicated by sepsis, is a rare yet highly lethal obstetric emergency characterized by synergistic endothelial dysfunction and coagulopathy. This condition presents significant diagnostic difficulties because of its similarities with severe preeclampsia and unusual sepsis. We present the case of a 30-year-old primigravida at 28 weeks’ gestation. She was hospitalized for oligohydramnios and mild hypertension, but quickly progressed to experiencing severe epigastric pain, fever, and multiorgan failure. Laboratory tests revealed thrombocytopenia (51 × 10⁹/L), microangiopathic hemolysis with schistocytes, hepatitis (aspartate aminotransferase 995 U/L), and hyperinflammation (interleukin-6 >500 pg/mL, procalcitonin 35.3 ng/mL). HELLP syndrome was confirmed after the exclusion of other thrombotic microangiopathies. Sepsis preceded the development of HELLP syndrome, with Klebsiella pneumoniae bacteremia and influenza A infection identified as the primary etiologies of sepsis. The multidisciplinary management involved emergent cesarean delivery, broad-spectrum antibiotics, plasma transfusion, and immunomodulation, resulting in progressive maternal recovery and neonatal survival despite premature birth. This case highlights the crucial importance of prompt sepsis screening in HELLP patients experiencing swift clinical decline. It also emphasizes the significance of protocolized interventions guided by Sequential Organ Failure Assessment and coordinated multidisciplinary efforts to reduce the combined risks of maternal-fetal mortality.

## Introduction

Hemolysis, Elevated Liver enzymes, and Low Platelets (HELLP) syndrome, a severe variant of preeclampsia, represents a critical obstetric emergency associated with significant maternal-fetal morbidity [1]. Sepsis complicating HELLP syndrome is driven by synergistic endothelial dysfunction and coagulopathy, which amplify systemic inflammation [2,3]. Although both conditions require urgent intervention, a clear diagnosis cannot be made using existing obstetric sepsis scores and MEOWS, and their diagnostic differentiation poses substantial challenges due to non-specific biomarker profiles [4], overlapping patterns of multi-organ failure [5], and pregnancy-induced immunological adaptations that obscure classical sepsis phenotypes [6].

Sepsis complicating HELLP syndrome represents a critical convergence of pathologies, with an estimated incidence of less than 1% of severe preeclampsia cases; however, precise epidemiological data remain scarce [7]. It predominantly manifests in the peripartum period, although its onset may be triggered by postpartum infections, invasive procedures, or dysregulated inflammatory cascades inherent to HELLP pathophysiology [2,6]. Early differentiation between HELLP progression and septic decompensation is crucial to mitigate synergistic multi-organ failure, yet this differentiation is profoundly challenged by overlapping biomarkers and pregnancy-adapted immune responses that obscure conventional systemic inflammatory response syndrome criteria [4,5]. Time-sensitive management necessitates protocolized plasma transfusion alongside empiric antimicrobial therapy, while diagnostic uncertainty often delays targeted interventions [8]. Therefore, multidisciplinary coordination among obstetrics, critical care, and microbiology teams is essential to optimize maternal survival and reduce neonatal sequelae, a paradigm underscored by this case yet inadequately addressed in current obstetric guidelines.

## Case presentation

Patient background, presentation, and initial findings

A 30-year-old primigravida (G1P0) with pre-pregnancy obesity (body mass index (BMI) 28.3 kg/m², total gestational weight gain 13 kg) presented to our hospital at 28 weeks and two days of gestation with newly diagnosed oligohydramnios. She denied fever, dizziness, visual disturbances, or dyspnea, but reported occasional palpitations. Her prenatal course had been uneventful before this visit, with routine screenings-including thyroid and renal function tests, oral glucose tolerance test, non-invasive prenatal testing, and fetal anatomical scans - all yielding normal results. No significant personal, surgical, family, or social medical history was noted. On physical examination, the patient had hypertension (139/102 mmHg) and bilateral lower extremity edema, with the gravid uterus showing a breech fetal presentation. Urinalysis revealed 1+ proteinuria, raising clinical suspicion for preeclampsia.

Prenatal surveillance before admission confirmed oligohydramnios (amniotic fluid index (AFI) 50 mm) and abnormal umbilical artery Doppler indices (systolic/diastolic ratio 4.44, resistance index 0.77). Baseline laboratory tests showed a hemoglobin level of 122 g/L, platelet count of 204 × 10⁹/L, and normal coagulation parameters. An elevated placental biomarker ratio (soluble fms-like tyrosine kinase-1 to placental growth factor (sFlt-1/PlGF) 330.45) further supported the diagnosis of preeclampsia. Fetal heart rate monitoring detected tachycardia with reduced variability, indicating chronic fetal distress. The patient was admitted to the obstetric ward for close monitoring and further evaluation.

On hospital day 3 (28 weeks and 5 days’ gestation), the patient developed sudden, severe epigastric pain radiating to the back, accompanied by tachypnea and a fever of 38.3 °C. Repeat vital sign measurements showed tachycardia (114 beats per minute) and persistent hypertension (141/101 mmHg). Abdominal examination revealed tenderness in the left upper quadrant, with no signs of peritoneal irritation.

Initial emergent laboratory tests indicated acute hepatitis (aspartate aminotransferase (AST) 147 U/L, alanine aminotransferase (ALT) 163 U/L) and elevated inflammatory markers (procalcitonin (PCT) 1.92 ng/mL, interleukin-6 (IL-6) >500 pg/mL). She was triaged as a high-priority obstetric emergency, with differential diagnoses including HELLP syndrome, sepsis, and intra-abdominal pathology.

Within hours, repeat laboratory testing demonstrated rapid, progressive deterioration: Mild anemia (hemoglobin 112 g/L), severe thrombocytopenia (platelets 51 × 10⁹/L), and microangiopathic hemolysis (schistocytes 12 per high-power field, lactate dehydrogenase (LDH) 1,000 U/L). Markedly elevated transaminases (AST 995 U/L, ALT 550 U/L) and hyperbilirubinemia (total bilirubin 56.9 μmol/L). Reduced fibrinogen (1.94 g/L). Sharply increased inflammatory markers (PCT 35.30 ng/mL, IL-6 1,396 pg/mL, C-reactive protein (CRP) 12.28 mg/L). Abdominal ultrasound of the liver showed no abnormalities, ruling out localized hepatic complications. Normal serum ammonia (32 μmol/L) and A disintegrin and metalloproteinase with a thrombospondin type 1 motif, member 13 (ADAMTS13) activity (78%) excluded acute fatty liver of pregnancy and thrombotic thrombocytopenic purpura, respectively. A Sequential Organ Failure Assessment (SOFA) score of 12 confirmed multi-organ failure. The patient was immediately transferred to the obstetric intensive care unit (ICU) for multidisciplinary management involving obstetricians, intensivists, hematologists, and infectious disease specialists (Table 1).

**Table 1:** Summary of key laboratory test results. Hb, hemoglobin; PLT, platelet count; WBC, white blood cell count; AST, aspartate aminotransferase; ALT, alanine aminotransferase; TBIL, total bilirubin; LDH, lactate dehydrogenase; FIB, fibrinogen; PCT, procalcitonin; IL-6, interleukin-6; CRP, C-reactive protein

Test item	Abbreviation	Admission baseline	Disease progression	Normal range
Hemoglobin	Hb	122	112	110-150 g/L
Platelet count	PLT	204 × 10⁹/L	51 × 10⁹/L	150-400 × 10⁹/L
White blood cell count	WBC	6.67 × 10⁹/L	6.37 × 10⁹/L	4.0-10.0 × 10⁹/L
Aspartate aminotransferase	AST	28 U/L	995 U/L	5-40 U/L
Alanine aminotransferase	ALT	32 U/L	550 U/L	7-56 U/L
Total bilirubin	TBIL	8.7 μmol/L	56.9 μmol/L	3.4-17.1 μmol/L
Lactate dehydrogenase	LDH	143 U/L	1000 U/L	120-250 U/L
Schistocytes (per HPF	-	-	12	0-1
Fibrinogen	FIB	3.8 g/L	1.94 g/L	2.0-4.0 g/L
Procalcitonin	PCT	1.92 ng/mL	35.30 ng/mL	<0.05 ng/mL
Interleukin-6	IL-6	7.14 pg/mL	1396 pg/mL	<7 pg/mL
C-reactive protein	CRP	3.19 mg/L	12.28 mg/L	<10 mg/L
Serum ammonia	-	-	32 μmol/L	11-45 μmol/L
ADAMTS13 Activity	-	-	78%	>60%
sFlt-1/PlGF Ratio	-	330.45	-	<38
Bacterial culture (blood)	-	-	Staphylococcus hominis + Klebsiella pneumoniae	No growth
Urine culture	-	-	Enterococcus faecalis	No growth

**Figure 1:**
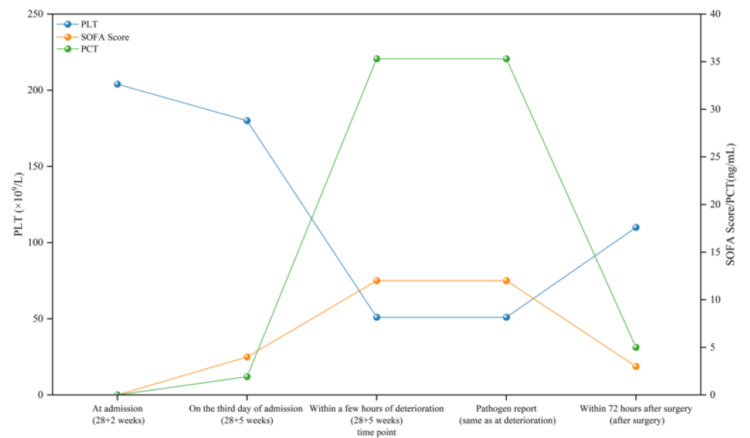
Timeline of cases of HELLP syndrome combined with sepsis. HELLP, hemolysis, elevated liver enzymes, and low platelet count; SOFA, Sequential Organ Failure Assessment; PLT, platelet count

Given the patient’s rapid clinical decline and multi-organ involvement, sepsis was prioritized for evaluation despite the lack of an initial focal infection site. Subsequent blood cultures confirmed bacteremia due to Staphylococcus hominis and Klebsiella pneumoniae, and further testing identified concurrent influenza A infection. Urine culture later detected Enterococcus faecalis, indicating a urinary tract infection as the likely source of bacteremia (Figure 1).

Emergency management and therapeutic intervention

Emergency management and therapeutic intervention were initiated due to maternal instability (Sequential Organ Failure Assessment score of 12) and non-reassuring fetal status, leading to an immediate cesarean delivery under general anesthesia. A male infant was delivered alive with Apgar scores of 3 and 8 at one and five minutes, respectively, and subsequently transferred to the neonatal intensive care unit. Postoperatively, the patient was treated with broad-spectrum antibiotics (meropenem) and clinically individualized intravenous immunoglobulin for sepsis. Additionally, plasma transfusion and platelet support were administered for coagulopathy, magnesium for seizure prophylaxis, and antihypertensives for preeclampsia.

Postpartum recovery and final diagnosis

**Figure 2:**
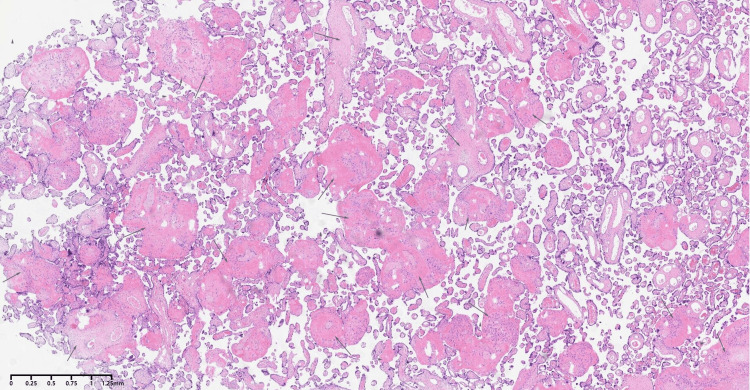
Placental pathological examination confirmed fibrinoid necrosis and infarction of the placental villi.

The patient was stabilized in the intensive care unit, with a gradual resolution of hepatic dysfunction, thrombocytopenia, and inflammatory markers over a period of 72 hours. Placental pathology confirmed extensive infarction and maternal vascular malperfusion. Final diagnoses included HELLP syndrome, sepsis secondary to bacteremia and influenza A, severe preeclampsia with placental insufficiency, and urinary tract infection as the source of bacteremia (Figure 2).

At the six-week postpartum follow-up, all of the patient’s laboratory parameters had returned to normal, with no long-term sequelae. The neonate (28 weeks + 5 days’ gestation; birth weight, 1,100 g) was promptly admitted to the neonatal intensive care unit (NICU) and received prophylactic surfactant therapy and nasal continuous positive airway pressure (nCPAP), 72-hour empiric antibiotic therapy (ampicillin and gentamicin) based on maternal infection status, and parenteral nutrition followed by fortified breast milk feeding support. No major complications occurred. Discharged at 32 weeks corrected gestation (weight 1,550 g) on full oral feeds without respiratory support, the infant achieved age-appropriate milestones at one-month follow-up, with no bronchopulmonary dysplasia or neurodevelopmental delay.

## Discussion

HELLP syndrome, a life-threatening variant of preeclampsia, presents significant diagnostic and therapeutic challenges when complicated by sepsis, an underreported yet high-risk obstetric scenario [1,8]. This case exemplifies the dangerous interplay between these conditions, highlighting the necessity for prompt recognition and multidisciplinary intervention to reduce catastrophic maternal and fetal outcomes. The overlapping pathophysiology of endothelial dysfunction and coagulopathy in both HELLP syndrome and sepsis [2,3] exacerbates diagnostic ambiguity, frequently delaying targeted management.

The classical triad of hemolysis, elevated liver enzymes, and thrombocytopenia is essential for the diagnosis of HELLP syndrome. However, sepsis introduces confounding variables; inflammatory biomarkers and indicators of organ failure display non-specific patterns that hinder early differentiation [4,5]. In this patient, the initial presentation of isolated oligohydramnios and mild hypertension masked an impending catastrophe. The subsequent onset of abrupt fever (38.3 °C), epigastric pain, and respiratory distress indicated systemic deterioration, yet the absence of clear infection foci complicated the timely diagnosis of sepsis. Notably, pregnancy-induced immunological modulation further diminished classic sepsis phenotypes [6], facilitating rapid progression to multi-organ dysfunction.

Pathophysiological convergence between HELLP syndrome and sepsis exacerbates microvascular injury. HELLP-associated platelet activation and hemolysis synergize with sepsis-driven disseminated intravascular coagulation, leading to consumptive coagulopathy, as evidenced by a significant decrease in platelet count (204 to 51 × 10⁹/L), an increase in D-dimer levels (0.59 to >3.96 mg/L), and fibrinogen depletion (1.94 g/L). Concurrently, sepsis-induced cytokine storms (IL-6 >500 pg/mL) exacerbate hepatic necrosis in HELLP, resulting in severe transaminitis (aspartate aminotransferase 995 U/L) and hyperbilirubinemia [2]. This bidirectional amplification highlights the vicious cycle that facilitates fulminant multi-organ failure within hours. The inability of the Obstetric Sepsis Score or MEOWS to distinguish between the progression of HELLP and septic decompensation may delay targeted interventions. In contrast, the standardized SOFA score, although not specifically designed for obstetrics, proved to be more objective and practical in our cases. It not only quantified severe sepsis with multiple organ dysfunction syndrome (with a score of 12) but also reflected the rapid deterioration of the disease through its sharp increase in the short term, serving as a core quantitative basis to demonstrate the causal relationship between infection and the progression of HELLP syndrome.

Plasma transfusion and emergent cesarean delivery are essential in managing HELLP syndrome, while sepsis necessitates simultaneous antimicrobial stewardship and hemodynamic support. A multidisciplinary approach guided by the Sequential Organ Failure Assessment score prioritized administering broad-spectrum antibiotics (meropenem) for culture-negative sepsis, expediting delivery to remove inflammatory triggers, and utilizing immunomodulation with intravenous immunoglobulin. Postpartum detection of Enterococcus faecalis urinary tract infection and Klebsiella pneumoniae bacteremia confirmed the sepsis diagnosis, underscoring the challenges of initial culture-negative presentations [7]. The positive maternal outcome following source control (delivery) and tailored antibiotics underscores the critical role of timely intervention in determining survival.

Diagnostic challenges emerged due to overlapping symptoms: early hypertension (139/102 mmHg) and proteinuria initially suggested isolated preeclampsia, while rising procalcitonin (1.92-35.3 ng/mL) was first attributed to HELLP-related inflammation. Critical differentiating features only manifested during clinical deterioration, including severe thrombocytopenia disproportionate to typical HELLP syndrome, lactate dehydrogenase elevation to 1,000 U/L, and microvascular hemolysis with schistocytes (ruling out thrombotic thrombocytopenic purpura). This case thus emphasizes the need to rigorously exclude sepsis in all HELLP syndrome patients with unexplained fever or rapid clinical decline, irrespective of biomarker specificity [5].

Supportive care nuances necessitate pregnancy-specific adaptations. Heparin thromboprophylaxis must balance the thrombotic risk associated with HELLP syndrome against the coagulopathy induced by sepsis, while fluid resuscitation requires careful monitoring for pulmonary edema in cases of capillary leak syndrome. Although corticosteroids are beneficial for fetal lung maturation in HELLP syndrome, their use in sepsis remains controversial and was withheld in this instance due to concerns regarding immunosuppression [8]. Continuous multi-parameter monitoring of lactate, platelets, and renal and liver function has proven essential for navigating therapeutic trade-offs.

Current knowledge gaps persist due to the rarity of HELLP-sepsis overlap. Most evidence is derived from case reports such as this one, highlighting the necessity for collaborative registries to refine diagnostic algorithms and treatment protocols [8,7]. Early delivery remains the definitive therapy for HELLP; however, sepsis complicates neonatal outcomes. In this case, preterm delivery at 28 weeks and a low Apgar score of 3 and 8 reflect the burdens of dual pathology.

Long-term vigilance is crucial for patients with HELLP-sepsis, as they are at increased risk of developing chronic hypertension, renal impairment, and experiencing sepsis recurrence in future pregnancies. Genetic counseling is recommended due to the possibility of inherited thrombophilias. The successful recovery of this patient highlights the importance of multidisciplinary preparedness, protocol-driven escalation, and postpartum source control in effectively managing this deadly combination.

## Conclusions

The co-occurrence of HELLP syndrome and sepsis presents a formidable diagnostic and therapeutic challenge. This case demonstrates that a high index of suspicion for concurrent sepsis is warranted in HELLP patients with rapid clinical deterioration, even without an obvious infectious source. Successful management hinges on a protocolized, multidisciplinary approach that includes prompt delivery, early administration of broad-spectrum antibiotics, and aggressive supportive care to address the synergistic endothelial and coagulation dysfunction. Future research should focus on adapting the SOFA score and Sepsis-3 criteria for the obstetric population, thereby facilitating early identification and targeted interventions for this susceptible cohort. Concomitant efforts should focus on developing pregnancy-specific diagnostic criteria for sepsis to enable timely recognition and intervention in this vulnerable patient population.
